# Increased expression of carbonic anhydrase I in the synovium of patients with ankylosing spondylitis

**DOI:** 10.1186/1471-2474-11-279

**Published:** 2010-12-08

**Authors:** Xiaotian Chang, Jinxiang Han, Yan Zhao, Xinfeng Yan, Shui Sun, Yazhou Cui

**Affiliations:** 1 National Laboratory for Bio-Drugs of Ministry of Health, Provincial Laboratory for Modern Medicine and Technology of Shandong, Research Center for Medicinal Biotechnology of Shandong Academy of Medical Sciences. Jingshi road 18877, Jinan, Shandong, 250062, P. R. China; 2 Orthopedic Surgery Center of Shandong Qianfoshan Hospital, Jinan, Shandong, P. R. China; 3 Orthopedic Surgery Center of Provincial Hospital of Shandong, Jinan, Shandong, P. R. China

## Abstract

**Background:**

One of the most distinctive features of ankylosing spondylitis (AS) is new bone formation and bone resorption at sites of chronic inflammation. Previous studies have indicated that the hyperplasia and inflammation of synovial tissues are significantly related to the pathogenic process of AS. The present study used a proteomic approach to identify novel AS-specific proteins by simultaneously comparing the expression profiles of synovial membranes from patients with AS, rheumatoid arthritis (RA) and osteoarthritis (OA).

**Methods:**

Synovial tissues were collected from the hip joints of patients with AS and knee joints of patients with RA or OA (n = 10 for each disease) during joint replacement surgery. Proteins extracted from the synovial tissues were separated by 2-D electrophoresis (2-DE), and the proteins with significantly increased expression in the AS samples were subjected to MALDI-TOF/TOF-MS analysis. The results were verified using western blotting and immunohistochemistry. Levels of the candidate proteins in synovial fluids from knee joints (n = 40 for each disease) were measured using ELISA.

**Results:**

The proteomic approach revealed significantly increased expression of carbonic anhydrase I (CA1) in the synovial membrane of patients with AS as compared with the RA and OA tissue samples. Immunohistochemistry and western blotting analysis confirmed the findings described above. The ELISA detected a higher level of CA1 in synovial fluids from patients with AS than those with OA. The mean value of the CA1 level was also higher in AS patients as compared with RA patients. This study also detected increased expression of alpha-1-antitrypsin in the synovial tissues from AS patients, which is in agreement with other reports.

**Conclusion:**

*In vitro *experiments by other groups indicated that CA1 catalyzes the generation of HCO_3_^- ^through the hydration of CO_2_, which then combines with Ca^2+ ^to form a CaCO3 precipitate. Calcification is an essential step of bone formation. Substantial evidence indicates that carbonic anhydrase also stimulates bone resorption. Hence, overexpression of CA1 in the synovial tissues of AS patients may promote improper calcification and bone resorption in AS.

## Background

Ankylosing spondylitis (AS) is a chronic inflammatory disease that can cause significant complications by affecting the sacroiliac joints and axial skeleton. AS is characterized by two key pathologic features: sacroiliac joint and spinal inflammation and new bone formation with possible bone fusion, usually in the axial skeleton [1,2]. However, the pathologic mechanism of AS, especially the molecular mechanism of ossification, remains largely unclear. Histopathological experiments demonstrated that severe forms of AS significantly correlate with villous chronic synovitis, including obliterating vascularitis, fibrosclerosis, necrosis and calcification of disintegrated synovial structures [3]. The global disease activity of AS significantly correlates with hyperplasia of the synovial membrane as well as with inflammatory infiltration of macrophages, especially the CD163+ subset, and polymorphonuclear leukocytes [4]. These studies revealed that pathological changes in the synovium of patients with AS are closely related to disease progression.

Proteomics is a powerful new tool for rheumatology research. Using this approach, Liu et al. demonstrated high expression of the haptoglobin precursor in the sera of patients with AS [5]. Write et al. detected upregulation of the beta subunit of the proteasome activator PA28 in AS monocytes [6]. In the present study, we performed a proteomic analysis of AS synovial membranes and compared the expression profile with profiles from rheumatoid arthritis (RA) and osteoarthritis (OA) synovial membranes. AS has some similar symptoms to RA and was clinically classified as RA many years ago [7]. OA is arthritic disease involving the degradation of joints including articular cartilage and the adjacent subchondral bone. Therefore, comparing the expression profiles of these three diseases could effectively filter out inflammatory proteins and metabolism-related proteins to discover protein biomarkers that are specific to AS.

## Methods

### Patients and sample collection

Synovial tissues were collected during joint replacement surgery from patients with AS (n = 10, 3 female; 24-54 years old, mean 34), RA (n = 10, 8 female; 30-65 years old, mean 51) and OA (n = 10, 4 female; 40-72 years old, mean 62). The AS tissues were collected from the hip joints of patients, and the RA and OA tissues were collected from the knee joints of patients. The samples were dissected from the connective tissues and immediately stored at -80°C until use. AS patients had an average disease duration of 7 years and were positive for the HLA-B27 antigen. Their symptoms fulfilled the modified New York criteria for AS [8]. The diagnosis of RA fulfilled the American College of Rheumatology criteria. The patients with RA had disease durations of 3 to 10 years and were classified as having erosive RA (Larsen class IV-V). They had high levels of C-reactive protein (6-192 mg/liter, mean 70 mg/liter), anti-CCP (92-394 U/ml) and RF (78-1280 U/ml). Patients with AS and RA took disease-modifying antirheumatic drugs (DMARDs) before surgery. Patients with AS, RA and OA were also medicated with non-steroidal anti-inflammatory drugs (NSAIDs), which help reduce the pain and swelling of the joints and decrease stiffness. Table 1 summarizes the epidemiological data.

**Table 1:** Clinical data on patients with AS, RA and OA

Diagnosis	**Patient No**.	Sex	Age (years)	HLA-B27	RF	anti-CCP U/ml	ESR (mm.h-1)	CRP (mg.dL-1)	Disease duration (years)	NSAIDs	DMARDs
AS	1	male	35	+	-	-	46	34	3	yes	yes
AS	2	male	29	+	-	-	7	6	12	yes	yes
AS	3	male	24	+	-	-	16	6	5	yes	yes
AS	4	male	31	+	-	-	45	6	6	yes	yes
AS	5	male	36	+	-	-	40	6	6	yes	yes
AS	6	male	37	+	-	-	24	5	8	yes	yes
AS	7	male	13	+	-	-	62	8	7	yes	yes
AS	8	female	48	+	-	-	58	10	5	yes	yes
AS	9	female	28	+	-	-	10	6	10	yes	yes
AS	10	female	54	+	-	-	12	6	10	yes	yes

RA	11	female	30	ND	100	209	80	6	4	yes	yes
RA	12	female	58	ND	160	279	72	192	9	yes	yes
RA	13	female	65	ND	496	218	66	153	7	yes	yes
RA	14	female	41	ND	78	168	39	10	3	yes	yes
RA	15	female	64	ND	200	169	61	10	5	yes	yes
RA	16	male	53	ND	107	171	55	20	3	yes	yes
RA	17	female	49	ND	160	227	50	96	4	yes	yes
RA	18	male	50	ND	1280	394	84	153	6	yes	yes
RA	19	female	53	ND	599	171	31	6	7	yes	yes
RA	20	female	48	ND	107	92	13	60	10	yes	yes

OA	21	male	40	ND	-	ND	10	6	6	yes	-
OA	22	female	62	ND	-	ND	68	48	10	yes	-
OA	23	female	53	ND	-	ND	20	6	8	yes	-
OA	24	female	60	ND	-	ND	22	6	8	yes	-
OA	25	male	66	ND	-	ND	24	10	6	yes	-
OA	26	female	72	ND	-	ND	24	6	5	yes	-
OA	27	male	65	ND	-	ND	5	10	11	yes	-
OA	28	male	72	ND	-	ND	8	6	13	yes	-
OA	29	male	68	ND	-	ND	24	6	7	yes	-
OA	30	male	67	ND	-	ND	10	20	6	yes	-

ND: no data available

Synovial fluids were aspirated from the joints of patients with AS (n = 40, 12 females; 26-54 years old, mean 35), RA (n = 40, 30 females; 23-73 years old, mean 44) and OA (n = 40, 16 females; 47-86 years old, mean 61). Patients with AS had disease durations of 3 to 8 years. Patients with RA had disease durations of 3 to 10 years. Patients with OA had disease durations of 3 to 13 years. The diagnosis is described above. They were medicated with NSAIDs and DMARDs.

All patients provided informed consent, and the study protocol was approved by the Ethics Committee of the Shandong Academy of Medical Sciences.

### 2-DE, protein spot collection and MALDI-TOF/TOF-MS analyses

Synovial tissues were homogenized and added to lysis buffer (7 M Urea, 2 M thiourea, 4% CHAPS, 2% IPG buffer, 65 mM DTT, 1 mM PMSF and Protease Inhibitor Cocktail). The extracted proteins were purified with a 2-D Clean-Up Kit (GE Healthcare), which improves the quality of 2-D electrophoresis results by removing interfering contaminants. The concentrations of the protein samples were determined with the 2-D Quant Kit (GE Healthcare). Cell lysates from AS, RA and OA synovial tissues with equal protein content were pooled. Isoelectric focusing (IEF) was applied to 24-cm IPG strips (pH 3-10 NL; GE Healthcare) using an Ettan IPGphor II IEF system (GE Healthcare). One hundred microgram of the protein sample was applied on IPG strips. After equilibration, the second dimension was performed on 12.5% SDS-polyacrylamide gels at a consistent power supply (with initial separation at a constant 0.2 w/gel for 1 hour followed by 17 w/gel at 25°C). The proteins were then stained with Coomassie Blue R350 (GE Healthcare) and scanned using a PowerLook 2100 XL scanner system (Umax). Gel images were analyzed with the Imagemaster 5.0 software package (GE Healthcare), and the spots with a 3-fold intensity change were excised from the gels for MALDI-TOF/TOF-MS analysis.

Gel spot pieces were destained in 50 mM ammonium bicarbonate/50% acetonitrile (ACN) buffer, dehydrated in 100% ACN for 15 minutes, and then dried by vacuum centrifugation. The dried pieces were added to modified trypsin buffer (Promega) (10 ng/μl in 25 mM NH_4_HCO_3_, pH 8.0) and incubated overnight at 37°C. Peptides were extracted with 5% trifluoroacetic acid (TFA)/50% ACN (v/v). After vacuum centrifugation, the lyophilized extract was redissolved in 0.1% TFA/30% ACN (v/v). Extracted peptides were mixed with an equal volume of α-cyano-4-hydroxycinamic acid matrix (α-CHCA) matrix (Sigma) and then spotted onto a standard 192-well plate for mass spectrometry analysis using an ABI 4700 Proteomics Analyzer MALDI-TOF/TOF mass spectrometer (ABI). Peptide mass fingerprinting (PMF) was acquired in a reflective mode, and at least two matched peaks were further validated by TOF-TOF analysis. The spectra data were searched against a human subset of the Swiss-Prot database for protein identifications using GPS explorer software.

### Western blot analysis

Two hundred micrograms of each sample from synovial tissues (n = 5 for each diseases) were homogenized in Cell Lysis Solution (Sigma) and centrifuged at 16,000 × g for 5 min at 4°C. The supernatant was collected after centrifugation, and the protein concentration was determined using the BCA protein assay kit (Pierce). Five micrograms of total protein were loaded and separated by sodium dodecyl sulfate-polyacrylamide gel electrophoresis (SDS-PAGE), transferred onto nylon membranes and probed with anti-human carbonic anhydrase I (CA1) antibody (Abcam, USA). The antibody was prepared by immunizing a goat with carbonic anhydrase I extracted from human erythrocytes. The manufacturer confirmed no cross-reactivity with other carbonic anhydrases. Immunoreactive signals were detected with alkaline phosphatase-conjugated secondary antibodies and visualized using a western Blotting Luminol Reagent (Amersham). Images of western blots were acquired on a Typhoon Trio (GE Healthcare). The quantification was conducted using ImageQuant5.2 software. Another membrane was prepared using the same protocol and probed with anti-GADPH antibody (Santa Cruz) to normalize for sample loading.

### Immunohistochemistry

Synovial tissues (n = 10 for each diseases) were fixed in 10% neutral buffered formalin for 12 hours at room temperature, embedded in paraffin and sectioned using standard procedures. Tissue sections were deparaffinized and rehydrated using standard procedures. To increase immunostaining intensity, the sections were heated at 95°C for 10 min in citrate buffer (0.01 M, pH 6.0). Sections were incubated with the primary antibody overnight at 4°C. Following incubation, tissue sections were washed three times for three minutes each in PBS and then processed with the UltraSensitive TM S-P kit (Maixin-Bio, China) according to the manufacturer's instructions. Immunoreactive signals were visualized using the DAB substrate, which stains the target protein a brown color. Tissue structure of the section was defined by counterstaining with hematoxylin.

### ELISA

The fluid samples were centrifuged at 3,000 × g for 10 min at 4°C to remove debris. Fluid samples were diluted 20-fold with 0.05 M carbonate-bicarbonate buffer (pH 9.6) and were used to coat 96-well ELISA microplates (Costar) by overnight incubation at 4°C. After a brief wash with PBS containing 0.1% Tween 20 (PBST), the plates were blocked with 5% non-fat dry milk for 1 hour at room temperature. The anti-CA1 antibody was diluted 1,000-fold with PBST, added to the plate, and incubated for 2 hours at room temperature. After washing with PBST, the plate was incubated with a 10,000-fold dilution of anti-goat IgG alkaline phosphatase-conjugated antibody (Sigma) for 30 min at room temperature. Following a wash with PBST, the signal was developed by adding Alkaline Phosphatase Yellow (pNPP) Liquid Substrate System For ELISA (Sigma). The absorbance of the reaction was measured at 405 nm with a plate reader (Synergy HT, Bio-Tek).

Statistical analysis of the data was performed using SPSS V.16 software (SPSS, USA). Median differences were tested with the Mann-Whitney U test. P values of less than 0.05 were considered significant. When three groups were compared, a Kruskal-Wallis test was conducted first.

## Results

### Expression of CA1 in synovial membranes

**Figure 1:**
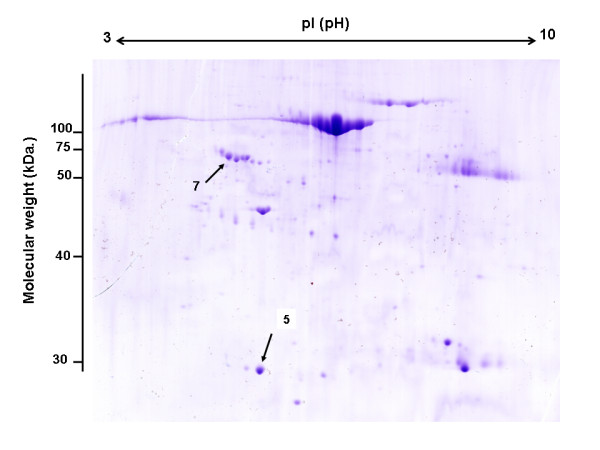
**Two-dimensional polyacrylamide gel electrophoresis of the total protein extracted from the synovial tissues of AS patients**. Protein was visualized by staining with colloidal Coomassie Blue. Protein spots indicated by numbers (#5 and #7) were identified by MALDI-TOF MS.

**Figure 2:**
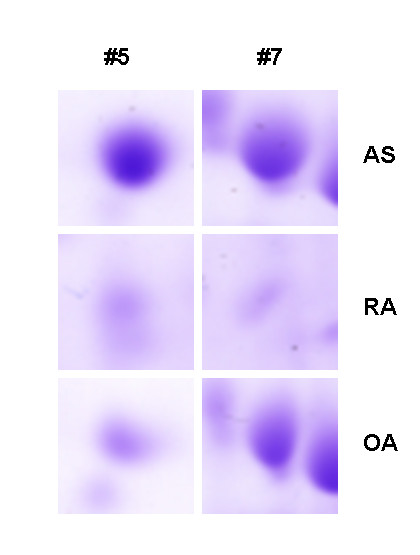
**Comparison of the AS, RA and OA synovial proteins indicated by numbers in Figure 1**. Protein spots (#5 and #7) from the AS synovial tissue had 3-fold higher expression levels than the matched spots from RA and OA synovium.

In the current study, comparative 2-DE was used to differentiate protein expression levels between the synovial membranes from AS and RA patients and AS and OA patients on a global scale. The experimental 2-DE gel patterns were highly reproducible over the three experiments. Following computational analysis, approximately 300 spots were visualized on each 2-DE gel. A 2-DE gel prepared with AS samples is shown in Figure 1. Two of the spots (#5 and #7) demonstrated 3-fold higher expression in the AS sample as compared with the RA and OA controls (Figure 2). Using MALDI-TOF MS, the spots with increased expression in AS synovial tissues were identified as carbonic anhydrase I (CA1) and alpha-1-antitrypsin precursor (A1AT). The detailed information about these identified proteins is shown in Table 2. By comparing the expression profiles of AS synovial membranes with those of RA, five other protein spots were detected with more than 3-fold greater expression. These proteins were identified as serotransferrin precursor, hemopexin precursor, apolipoprotein A-I precursor, dedicator of cytokinesis protein 2 and intersectin 1. By comparing the expression profiles of AS synovial membranes with those of OA, 11 protein spots were detected with more than 3-fold greater expression. These proteins were identified as Ig gamma-1 chain C region, dynein heavy chain, fibrinogen gamma chain precursor, catalase, SH3 and multiple ankyrin repeat domains protein 2, haptoglobin precursor, DNA-dependent protein kinase catalytic subunit, apolipoprotein A-I precursor, serotransferrin precursor, microtubule-actin crosslinking factor 1 isoform 4, and intersectin 1. However, these proteins were also expressed at increased levels in the synovial tissues of RA patients or in the synovial tissues of OA patients, and they were not specifically expressed at significantly increased levels in the synovial tissues of AS patients. Any differentially expressed spots demonstrating a less than 3-fold change were not addressed in the present study.

**Table 2:** Identification of spots with significant over-expression in AS synovial membrane by proteome analysis

spot	protein name	MW(kDa.) theoretical	pl theoretical	Peptide count	Protein score	Intensity matched %	protein species score C.I.%
#5	carbonic anhydrase 1	28778.4	6.63	9	135	80.75	100
#7	alpha-1-antitrypsin precursor	46878.1	5.37	10	133	84.79	100

MW, molecular weight; pl, isoelectric point.

**Figure 3:**
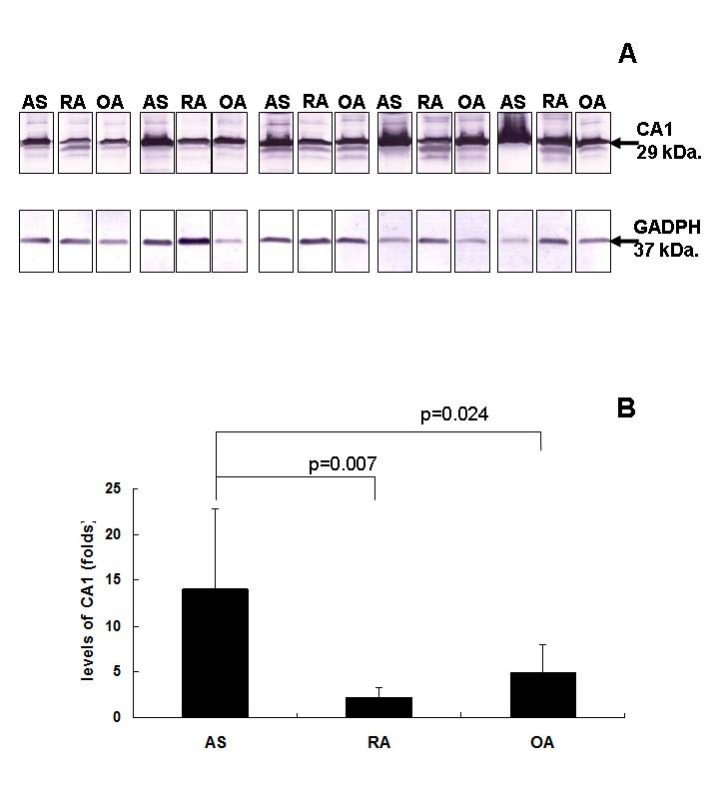
**Western blot analysis of CA1 expression in synovial membranes from AS, RA and OA patients**. (A) Western blotting of CA1 in AS, RA and OA synovial tissues (n = 5 for each disease). The molecular weight is indicated with arrows. Sample loading was normalized using GADPH. (B) The CA1 signal was normalized to the GADPH signal. The expression levels are expressed as the mean ± SEM. CA1 was expressed at a significantly higher level in AS synovial tissue than in RA and OA synovial tissues.

Western blot analysis was performed with antibodies against CA1. Using GADPH as a reference, CA1 showed significantly higher levels of expression in the synovial membranes of AS patients than in samples from RA and OA patients. The high level of expression was observed in all of five synovial membranes tested. Among the five OA synovial samples, only one sample had a relatively high CA1 expression. All of the RA synovial samples showed low levels of expression (Figure 3A and 3B). A similar result was also obtained using β-actin as a reference.

**Figure 4:**
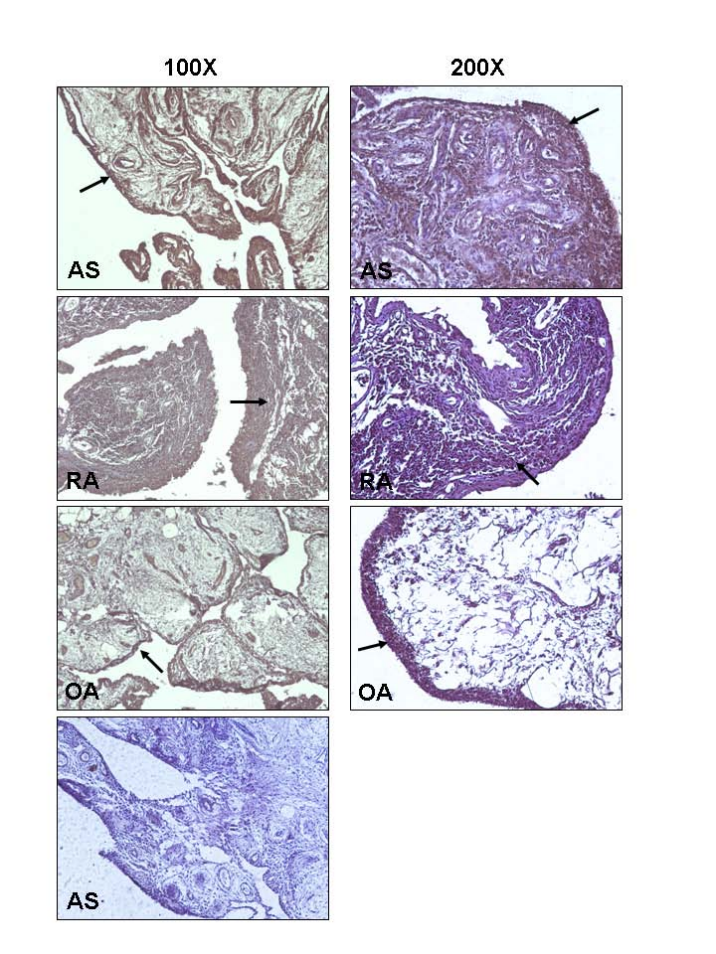
**Immunodetection of CA1 in the synovial membranes of AS, RA and OA patients**. CA1 was significantly detected with a strong immunosignal in AS synovial tissue. Original magnification: 100X and 200X. The picture at the bottom is a negative control prepared with AS synovial tissue that was not treated with primary antibody.

Immunohistochemistry detected CA1 expression in all AS synovial tissues (100%) and yielded high intensity signals. The CA1 immunosignal was observed in the lining layer, the endothelial cells around the small blood vessel and some fibroblast-like cells in the synovial membranes of AS patients. On the other hand, CA1 was detected in the thick lining layer of four samples of synovial membranes from RA patients (40%), but the signal intensity was relatively low and appeared as a smear. CA1 was detected in six samples of synovial membranes from OA patients (60%); the enzyme was detected in the upper lining within the thin layer of cells. The tissue distribution of CA1 is shown in Figure 4. The result is in agreement with the western blot analysis.

### Expression of CA1 in synovial fluid

**Figure 5:**
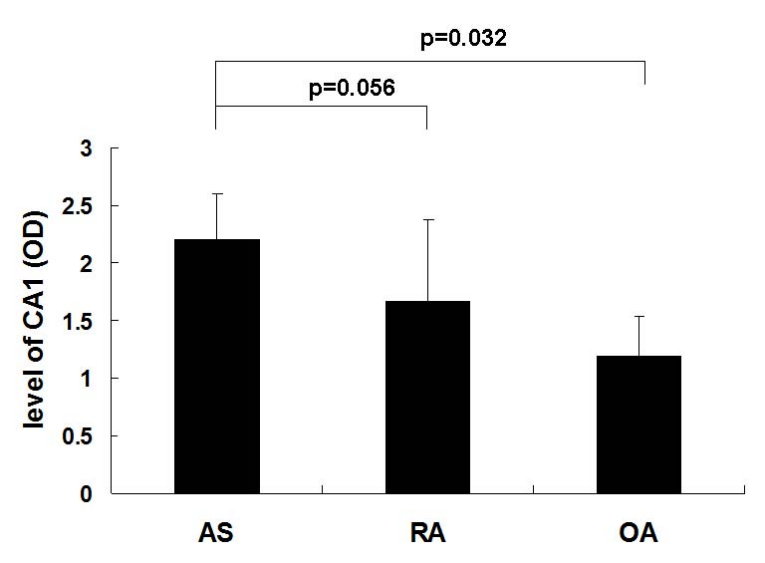
**Detection of CA1 level in synovial fluids by ELISA**. Levels are represented as O.D. values obtained from the absorbance at 405 nm. The results are expressed as the mean ± SEM. Expression of CA1 was higher in AS samples than in OA samples.

ELISA was used to measure the levels of CA1 in the synovial fluids from patients with AS, RA and OA at the chronic inflammation stage. CA1 levels were significantly elevated in AS fluids as compared with the OA samples (p = 0.032). Compared to the average level of the OA samples, fluid samples from AS patients had a two-fold or greater increase in CA1 expression in 31 samples (77.5%). Thirty-five of the AS samples (87.5%) had relatively high levels of CA1 (O.D. > 2.0). This level did not significantly change among OA samples, and only two OA samples had higher levels of CA1 (O.D. > 2.0). Among the 40 RA synovial fluid samples, 12 samples had higher levels of CA1 (30%, O.D. > 2.0). The mean levels of CA1 were higher in AS fluids than in RA fluids, although there was no statistically significant difference (p = 0.056). The ELISA result is shown in Figure 5.

## Discussion

In the present study, a proteomic approach exclusively identified the increased expression of CA1 in AS synovial membranes as compared with RA and OA samples. The increased expression of CA1 was confirmed by individually investigating synovial samples from each disease using western blot analysis. Immunohistochemistry also located CA1 in the AS synovial membranes with a strong signal density. Furthermore, the ELISA detected higher levels of CA1 in the synovial fluid from patients with AS compared to the OA samples. The mean value of the CA1 level is also higher in AS patients as compared to RA patients. The CA1 in the synovial fluid may be secreted by the AS synovial membranes.

Samples for each disease condition were pooled for 2-DE proteomics, which is regarded as the best practice in many studies to effectively find common features of diseased tissues under variable conditions. Under normal circumstances, proteomic research conducts a comparison of tissue samples from the two diseases, and it can consequently obtain many protein spots with specific expressions. In our study, we simultaneously compared the samples from three arthritic diseases, and we therefore obtained fewer but unique for the disease. Additionally, a significantly increased expression level of the alpha-1-antitrypsin (A1AT) precursor, which is an important plasma inhibitor of serine proteases, was detected in the synovial tissues of AS patients using a proteomic approach. Some reports have demonstrated the involvement of A1AT in the pathogenesis of AS. Increased levels of the complex of immunoglobulin with A-alpha 1 antitrypsin have been detected in the serum of AS patients and were associated with a clinical index of the disease [9,10]. Identifying A1AT in the synovial membrane of AS patients confirmed the feasibility and reliability of our protocol.

Tilleman et al. investigated the cytosolic proteome of inflamed synovial tissue and validated the feasibility of this proteomic analysis by identifying proteins that were differentially expressed between AS, RA and OA. They collected synovial biopsy samples from 18 patients undergoing needle arthroscopy for knee synovitis associated with AS (n = 6) and RA (n = 6) and for joint effusion of the knee associated with OA (n = 6). Following 2-DE, protein expression patterns were statistically analyzed and used for hierarchical cluster analysis. Proteins of interest were identified by matrix-assisted laser desorption/ionization- and electrospray ionization-mass spectrometry. By identifying proteins that were differentially expressed between AS and RA and AS and OA (P < 0.01), they finally detected higher expression of fructose bisphosphate aldolase A and alpha-enolase in AS synovial tissues than in OA synovial tissues. They also detected a higher level of calgranulin A myeloid-related protein-8 in the RA and AS samples than in the OA samples [11]. In our study, we conducted a proteomic analysis with whole synovial membranes of hip joints from AS patients as opposed to synovial biopsy samples taken by needle arthroscopy from the knee joint of the patients. Additionally, we identified novel AS-specific proteins by simultaneously comparing the expression profiles of synovial membranes from patients with AS, RA and OA. We thus detected especially high expression of CA1 in the synovial tissues of AS patients. Because our patients and their patients all received treatment with NSAIDs and DMARDs before surgery, the medical treatment could not contribute to the differences in the findings of our study and their study.

CA1 catalyzes the hydration of carbon dioxide and forms bicarbonate [12]. Carbonic anhydrases (CA) participate in the physiological and pathological activities of calcification and mineralization [13]. Parissa et al. investigated the effect of CA1 on the hydration of CO_2 _and the formation of calcium carbonate. They conducted an *in vitro *assay using a reaction mixture containing bovine CA1 and calcium chloride in Tris buffer. After CO_2 _was added, CA1 enhanced the hydration reaction, and the hydration reaction rate increased with both the enzyme concentration and temperature. Furthermore, they found that CA1 promotes the formation of CaCO_3 _and the precipitant quickly settles [14]. Ramana et al. also detected CaCO_3 _deposition in a calcium chloride solution saturated with CO_2 _in the presence of carbonic anhydrase purified from *Citrobacter freundii*. They observed a sharp decrease in CaCO_3 _formation with the addition of EDTA and acetazolamide, inhibitors of carbonic anhydrase [15]. These results indicated that CA1 not only enhances the hydration reaction of CO_2_, but it also promotes the combining of bicarbonate with calcium to form the solid precipitant of calcium carbonate. Therefore, overexpression of CA1 in the synovium of AS patients may lead to CaCO_3 _deposition in diseased tissues. One of the most distinctive features of AS is new bone formation at sites of chronic inflammation [2]. Bone is mainly composed of calcium phosphate and calcium carbonate [16]. In the physiological milieu, a counterbalancing inhibition is required to prevent inappropriate formation of insoluble crystals of calcium salt [17,18]. Increased CA1 expression in the synovium of AS patients may lead to improper mineralization by accelerating calcium salt deposition.

Increased bone resorption is a characteristic of AS [19,20]. Using an in vitro neonatal mouse calvarial culture system, Hall et al. found that carbonic anhydrase activity enhanced the stimulation of prostaglandin E_2 _for resorption, which indicates that carbonic anhydrase is a necessary component of the osteoclastic bone resorptive mechanism [21]. Two years later, the group found that the carbonic anhydrase inhibitor acetazolamide inhibited bone resorption [22]. Nolan et al. also found that carbonic anhydrase inhibitors including acetazolamide, ethoxzolamide, methazolamide, and dichlorphenamide reduced paw edema and attenuated joint deterioration in rats with adjuvant arthritis. They suggested that the carbonic anhydrase inhibitors played an antiarthritic role by inhibiting bone resorption [23]. These investigations demonstrated a functional role for carbonic anhydrase in the mediation of hormone-stimulated bone resorption [24]. Overexpression of CA1 in the synovium of AS patients may be involved in bone resorption of the disease. Additionally, studies have shown a high incidence of uveitis in patients with AS, and approximately 40% of patients with uveitis are also HLA-B27 positive [25,26]. Acetazolamide, inhibitor of CA1, is often used for the medical treatment of uveitis [27]. The high expression levels of CA1 in the synovial tissues of patients with AS may partially explain the strong association of the two diseases.

Most studies on AS focus on bone and bone development of the disease, although some studies had demonstrated that the global disease activity of AS significantly correlates with hyperplasia of the synovial membrane. Our results suggest that elevated expression of CA1 in the diseased synovial tissues plays a role in new bone formation and bone resorption. However, the importance of CA1 in AS processes is still unknown.

## Conclusions

The current study detected increased expression of CA1 in the synovial tissue of AS patients. It has been demonstrated that CA1 can lead to CaCO_3 _precipitants. Substantial evidence has indicated that carbonic anhydrase is also involved in bone resorption. These results suggest that the overexpression of CA1 in the synovium of AS patients may accelerate calcification and bone resorption, which are two essential processes for new bone formation. This finding may be helpful in understanding the pathogenic mechanism of AS.

## List of abbreviations

AS: Ankylosing spondylitis; RA: rheumatoid arthritis; OA: osteoarthritis; 2-DE: 2-D electrophoresis; CA1: carbonic anhydrase I.

## Competing interests

The authors declare that they have no competing interests.

## Authors' contributions

XC designed the study, executed data analysis and prepared the manuscript. YZ performed the immunohistochemistry, western blotting and ELISA. XY and SS recruited the patients and collected the synovial membranes and synovial fluids. YC and JH executed the proteomic study. All authors read and approved the final manuscript.

## Pre-publication history

The pre-publication history for this paper can be accessed here:

http://www.biomedcentral.com/1471-2474/11/279/prepub

## References

[B1] de Vlam K Lories RJ Luyten FP Mechanisms of pathologic new bone formation Curr Rheumatol Rep 2006 8 332 337 10.1007/s11926-006-0061-z 16973105

[B2] Zhang X Aubin JE Inman RD Molecular and cellular biology of new bone formation: insights into the ankylosis of ankylosing spondylitis Curr Opin Rheumatol 2003 15 387 393 10.1097/00002281-200307000-00004 12819465

[B3] Chen WS Chen CH Lin KC Tsai CY Liao HT Wang HB Chen YK Yang AH Chen TC Chou CT Immunohistological features of hip synovitis in ankylosing spondylitis with advanced hip involvement Scand J Rheumatol 2009 38 154 155 10.1080/03009740802409504 19165649

[B4] Baeten D Kruithof E De Rycke L Boots AM Mielants H Veys EM De Keyser F Infiltration of the synovial membrane with macrophage subsets and polymorphonuclear cells reflects global disease activity in spondyloarthropathy Arthritis Res Ther 2005 7 R359 369 10.1186/ar1501 15743484 PMC1065336

[B5] Liu J Zhu P Peng J Li K Du J Gu J Ou Y Identification of disease-associated proteins by proteomic approach in ankylosing spondylitis Biochem Biophys Res Commun 2007 357 531 536 10.1016/j.bbrc.2007.03.179 17434140

[B6] Wright CA Edelmann M Digleria K Kollnberger S Kramer H McGowan S McHugh K Taylor S Kessler BM Bowness P Ankylosing spondylitis monocytes show upregulation of proteins involved in inflammation and the Ubiquitin Proteasome pathway Ann Rheum Dis 2009 68 1626 1632 10.1136/ard.2008.097204 18952638

[B7] Helliwell PS The semeiology of arthritis: discriminating between patients on the basis of their symptoms Ann Rheum Dis 1995 54 924 926 10.1136/ard.54.11.924 7492243 PMC1010044

[B8] van der Linden S Valkenburg HA Cats A Evaluation of diagnostic criteria for ankylosing spondylitis: a proposal for modification of the New York criteria Arthritis Rheum 1984 27 361 368 10.1002/art.1780270401 6231933

[B9] Struthers GR Lewin IV Stanworth DR IgA-alpha 1 antitrypsin complexes in ankylosing spondylitis Ann Rheum Dis 1989 48 30 44 10.1136/ard.48.1.30 2784305 PMC1003671

[B10] Davis MJ Dawes PT Beswick E Lewin IV Stanworth DR Sulphasalazine therapy in ankylosing spondylitis: its effect on disease activity, immunoglobulin A and the complex immunoglobulin A-alpha-1-antitrypsin Br J Rheumatol 1989 28 410 413 10.1093/rheumatology/28.5.410 2571386

[B11] Tilleman K Van Beneden K Dhondt A Hoffman I De Keyser F Veys E Elewaut D Deforce D Chronically inflamed synovium from spondyloarthropathy and rheumatoid arthritis investigated by protein expression profiling followed by tandem mass spectrometry Proteomics 2005 5 2247 2257 10.1002/pmic.200401109 15846842

[B12] Sterling D Reithmeier RA Casey JR Carbonic anhydrase: in the driver's seat for bicarbonate transport JOP 2001 2 165 170 11875254

[B13] Supuran CT Carbonic anhydrases--an overview Curr Pharm Des 2008 14 603 614 10.2174/138161208783877884 18336305

[B14] Parissa M Koorosh A Nader M Investigating the Application of Enzyme Carbonic Anhydrase for CO_2 _sequestration purposes Ind Eng Chem Res 2007 46 921 926 10.1021/ie060287u

[B15] Ramanan R Kannan K Sivanesan SD Mudliar S Kaur S Tripathi AK Chakrabarti T Bio-sequestration of carbon dioxide using carbonic anhydrase enzyme purified from Citrobacter freundii World Journal of Microbiology and Biotechnology 2009 25 981 987 10.1007/s11274-009-9975-8

[B16] Boskey AL Posner AS Bone structure, composition, and mineralization Orthop Clin North Am 1984 15 597 612 6387574

[B17] Anderson HC Mechanism of mineral formation in bone Lab Invest 1989 60 320 330 2648065

[B18] Provot S Schipani E Molecular mechanisms of endochondral bone development Biochem Biophys Res Commun 2005 328 658 665 10.1016/j.bbrc.2004.11.068 15694399

[B19] Schett G Bone formation versus bone resorption in ankylosing spondylitis Adv Exp Med Biol 2009 649 114 121 full_text 19731624 10.1007/978-1-4419-0298-6_8

[B20] Grisar J Bernecker PM Aringer M Redlich K Sedlak M Wolozcszuk W Spitzauer S Grampp S Kainberger F Ebner W Smolen JS Pietschmann P Ankylosing spondylitis, psoriatic arthritis, and reactive arthritis show increased bone resorption, but differ with regard to bone formation J Rheumatol 2002 29 1430 1436 12136902

[B21] Hall GE Kenny AD Role of carbonic anhydrase in bone resorption induced by prostaglandin E2 in vitro Pharmacology 1985 30 339 347 10.1159/000138088 3925470

[B22] Hall GE Kenny AD Role of carbonic anhydrase in bone resorption: effect of acetazolamide on basal and parathyroid hormone-induced bone metabolism Calcif Tissue Int 1987 40 212 218 10.1007/BF02556624 3034387

[B23] Nolan JC Gathright CE Radvany CH Barrett RJ Sancilio LF Carbonic anhydrase inhibitors are antiarthritic in the rat Pharmacol Res 1991 24 377 383 10.1016/1043-6618(91)90042-V 1805191

[B24] Pierce WM Jr Waite LC Bone-targeted carbonic anhydrase inhibitors: effect of a proinhibitor on bone resorption in vitro Proc Soc Exp Biol Med 1987 186 96 102 3628257 10.3181/00379727-186-42590a

[B25] Braun J Sieper J Early diagnosis of spondyloarthritis Nat Clin Pract Rheumatol 2006 2 536 545 10.1038/ncprheum0296 17016479

[B26] Chang JH McCluskey PJ Wakefield D Acute anterior uveitis and HLA-B27 Surv Ophthalmol 2005 50 364 388 10.1016/j.survophthal.2005.04.003 15967191

[B27] Cox SN Hay E Bird AC Treatment of chronic macular edema with acetazolamide Arch Ophthalmol 1988 106 1190 1195 3415543 10.1001/archopht.1988.01060140350030

